# Alpha-glucosidase inhibitor use is associated with decreased colorectal neoplasia risk in patients with type 2 diabetes mellitus receiving colonoscopy: a retrospective study

**DOI:** 10.18632/oncotarget.18416

**Published:** 2017-06-08

**Authors:** Yohei Horibe, Seiji Adachi, Tomohiko Ohno, Naoe Goto, Mitsuru Okuno, Midori Iwama, Osamu Yamauchi, Takao Kojima, Koshiro Saito, Takashi Ibuka, Ichiro Yasuda, Hiroshi Araki, Hisataka Moriwaki, Masahito Shimizu

**Affiliations:** ^1^ Department of Gastroenterology and Internal Medicine, Gihoku Kosei Hospital, Yamagata, 501-2105, Japan; ^2^ Division for Regional Cancer Control, Gifu University Graduate School of Medicine, Gifu, 501-1194, Japan; ^3^ Department of Gastroenterology and Internal Medicine, Gifu University Graduate School of Medicine, Gifu, 501-1194, Japan

**Keywords:** type 2 diabetes mellitus, colorectal neoplasia, risk factor

## Abstract

**Purpose:**

The purpose of this study was to clarify the factors that influence the incidence of colorectal neoplasia in patients with type 2 diabetes mellitus (DM).

**Study Design and Setting:**

Among a total of 1176 patients who underwent total colonoscopy at our hospital, we retrospectively analyzed 168 patients with type 2 DM. Univariate and multivariate logistic regression analyses were then performed to identify the risk factors associated with colorectal neoplasia.

**Results:**

A multivariate analysis of these patients demonstrated that male gender (odds ratio [OR] = 4.04, 95% confidence interval [CI] = 1.67–10.37, *p* = 0.002), taking statins (OR = 4.59, 95% CI = 1.69–13.43, *p* = 0.003), taking alpha glucosidase inhibitor (α-GI) (OR = 0.35, 95% CI = 0.13–0.87, *p* = 0.023) and taking low-dose aspirin (LDA) (OR = 0.32, 95% CI = 0.10–0.95, *p* = 0.040) were independent factors associated with an increased (male gender and statins) or decreased (α-GI and LDA) risk of colorectal neoplasia.

**Conclusions:**

While male gender and taking statins are risk factors, taking α-GI as well as LDA may reduce the risk of colorectal neoplasia in patients with type2 DM.

## INTRODUCTION

Colorectal cancer (CRC) is a major global healthcare problem, including in Japan; the mortality rate of patients with CRC is twice that of 20 years ago. In Japan, this malignancy has become the third leading cause of cancer death in males and the leading cause of cancer death in females (http://www.mhlw.go.jp/english/) [1]. The incidence of CRC is still high in highly industrialized countries, and the number of CRC cases has increased in many developing countries that have undergone rapid economic transformations and adopted a Western lifestyle [2].

Because CRC usually arises from colorectal adenoma, the early detection and removal of colorectal adenoma by endoscopy can help avoid CRC-related death [3]. The prevention of colorectal adenoma development by determining the environmental risk factors and identifying high-risk patients might also be significant in reducing the rate of death caused by CRC. Accumulating evidence shows that a Western diet (imbalanced diet), sedentary lifestyle, alcohol consumption, tobacco use, lack of physical activity, sleep deprivation and obesity are significant risk factors for CRC [4–6]. Among these risk factors, obesity and its related medical problems, especially type 2 diabetes mellitus (DM), are well known to play a critical role in the development of CRC.

Several pathophysiological mechanisms have been shown to be involved in the correlation between type 2 DM and colorectal carcinogenesis [7], including the occurrence of insulin resistance and the induction of chronic inflammation [8, 9]. The effects of dietary- and lifestyle-related risk factors on CRC are suggested to be mediated through hyperinsulinemia [4], which occurs in most type 2 DM patients [10, 11]. Exogenous insulin injection stimulates the growth of CRC precursors in animal models [12, 13]. A recent prospective study revealed a significant relationship between the development of CRC and insulin use in patients with type 2 DM [14]. A systematic review and meta-analysis also showed that insulin use is a risk factor for CRC [15].

It is well-reported that several dietary factors, such as vitamins, green tea catechin and certain kinds of vegetables and fruits, can exert chemopreventive properties against colorectal neoplasia [16–20]. In a preliminary human trial, supplementation with green tea catechin, which can suppress diabetes-related colorectal carcinogenesis in mice by improving hyperinsulinemia [21], successfully prevented the development of colorectal adenomas [22]. Furthermore, several preclinical trials have shown that conventional anti-diabetic medications may modify the risk of some cancers, including CRC [8, 23, 24]. For instance, metformin, an anti-diabetic agent, is expected to prevent the development of colorectal neoplasms [25]. In addition to anti-diabetic drugs, several studies have revealed the correlation between a decreased risk of colorectal tumorigenesis and current medications for lifestyle-related diseases, such as hypertension and dyslipidemia [26–29]. These reports suggest that the evaluation of medications might be useful for identifying subjects at high risk for developing colorectal neoplasia.

In the present study, we investigated the factors that influence the incidence of CRC in patients with type 2 DM. These factors included the patient’ characteristics, medical history, family history, habits (smoking and alcohol intake) and medications associated with lifestyle-related disease, such as type 2 DM, hypertension, dyslipidemia and ischemic disease due to atherosclerosis. These factors are commonly confirmed with a medical interview and can therefore useful in a clinical setting. The purpose of this study was to identify diabetic patients at a higher or lower risk for CRC development with a focus on the factors described above.

## RESULTS

### Patient flowchart and characteristics of patients with or without type 2 DM

**Figure 1:**
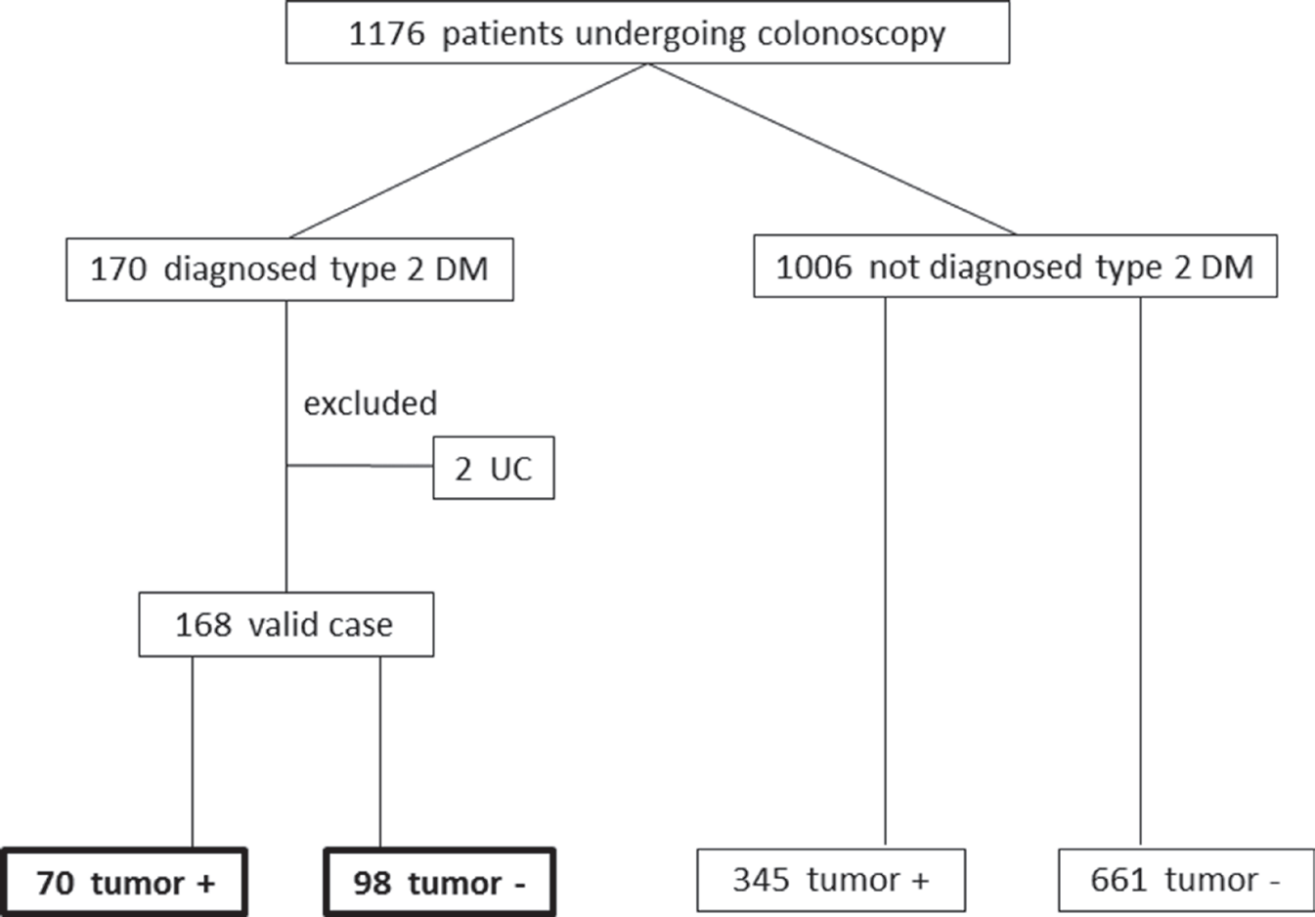
Patient flowchart DM: diabetes mellitus. UC: ulcerative colitis.

The patient flowchart is shown in Figure 1. Among the 1176 patients who were enrolled in this study, the 170 who met the diagnostic criteria of the Japanese Diabetes Society [30] or who were prescribed anti-diabetic medications were diagnosed with type 2 DM. Among them, two cases were excluded because of the presence of UC. Thus, 168 diabetic patients were ultimately evaluated. Colorectal neoplasia was detected by TCS in 70 (41.7%) of these type 2 DM patients. Colorectal neoplasia was detected in 345 of the 1006 (34.3%) non-diabetic patients in this study. As shown in Table 1, the statistical analysis demonstrated significant differences between the diabetic and non-diabetic patients with regard to the mean age (*p* < 0.001), the proportion of patients with a higher BMI (≥ 25, *p* < 0.001) and complication with hypertension (*p* < 0.001) and dyslipidemia (*p* = 0.002). The incidence of colorectal neoplasia tended to be higher in diabetic patients than in non-diabetic patients; however, the difference did not reach statistical significance (*p* = 0.064).

**Table 1:** Baseline characteristics of the patients with or without type 2 DM

	DM (*n* = 168)	non DM (*n* = 1006)
Age, mean ± SD	71.6 ± 10.1 ^*^	65.2 ± 15.4
Gender, male	111 (65.3%)	585 (58.4%)
BMI (≥ 25 kg/m^2^)	55 (34.4%) ^*^	157 (16.8%)
Hypertension	98 (58.3%) ^*^	371 (36.9%)
Dyslipidemia	60 (35.7%) ^*^	238 (23.7%)
Alcohol use	57 (33.9%)	351 (31.3%)
Smoking	39 (23.2%)	224 (22.3%)
Incidence of colorectal neoplasia	70 (41.7%)	345 (34.3%)
HbA1c (%), mean ± SD	7.3 ± 2.2	N/A

DM: diabetes mellitus, BMI: body mass index, SD: standard deviation.

^*^:Significant difference between the groups (*p* < 0.05).

### A comparison of the characteristics between type 2 diabetic patients with or without colorectal neoplasia

Table 2 shows the comparison of the clinical characteristics between diabetic patients with or without colorectal neoplasia detected by TCS. There were no significant differences in the median age, the BMI, the HbA1c values or the rate of positive FIT between the tumor group (*n* = 70) and the non-tumor group (*n* = 98). The proportion of men in the tumor group was significantly higher than in the non-tumor group (77.1% vs. 56.1%, *p* = 0.005). There were no significant differences between the two groups with regard to the rates of complication with hypertension and dyslipidemia, alcohol consumption or smoking.

**Table 2:** Comparison of the clinical characteristics in patients with or without colorectal neoplasia

	Presence of colorectal neoplasia		
	Yes (*n* = 70)	No (*n* = 98)	
Characteristic	% (*n*)	% (*n*)	*p*-value
Age (years), mean ± SD	72.2 ± 9.3	71.1 ± 10.7	*p* = 0.455
Gender, male (%)	77.1 (54)	56.1 (55)	*p* = 0.005
BMI (≥ 25 kg/m^2^)	29.4 (20)	35.7 (34)	*p* = 0.420
HbA1c, mean ± SD	7.02 ± 1.6 (18)	7.50 ± 2.4 (37)	*p* = 0.455
Positive of FIT (%)	63.7 (14)	53.3 (16)	*p* = 0.458
Complications			
Hypertension	58.6 (41)	57.1 (56)	*p* = 0.853
Dyslipidemia	37.1 (26)	34.7 (34)	*p* = 0.744
Habits			
Alcohol use	37.1 (26)	30.6 (30)	*p* = 0.376
Smoking	30 (21)	18.4 (18)	*p* = 0.078
Medications			
LDA	8.6 (6)	18.4 (18)	*p* = 0.079
ADPR	4.3 (3)	13.3 (13)	*p* = 0.063
PDEIII-I	1.4 (1)	4.1 (4)	*p* = 0.403
Anti-coagulant	8.6 (6)	2.0 (2)	*p* = 0.068
Sulfonylureas	28.6 (20)	23.5 (23)	*p* = 0.455
Biguanide	18.6 (13)	20.4 (20)	*p* = 0.768
Tiazoridine	17.1 (12)	10.2 (10)	*p* = 0.189
α-GI	12.9 (9)	27.6 (27)	*p* = 0.023
Glinide	4.3 (3)	1.0 (1)	*p* = 0.309
DPP4	48.6 (34)	44.9 (44)	*p* = 0.638
Insulin	7.1 (5)	9.2 (9)	*p* = 0.780
ACEI	4.3 (3)	6.1 (6)	*p* = 0.736
ARB	37.1 (26)	32.7 (32)	*p* = 0.546
Ca-blocker	42.9 (30)	34.7 (34)	*p* = 0.283
β-blocker	5.7 (4)	7.1 (7)	*p* = 0.764
α-blocker	8.6 (6)	9.2 (9)	*p* = 0.891
Statin	35.7 (25)	19.4 (19)	*p* = 0.018
Fibrate	2.9 (2)	4.1 (4)	*p* = 0.673
NSAIDs	20.0 (14)	20.4 (20)	*p* = 0.948

DM: diabetes mellitus, SD: standard deviation, FIT: fecal immunochemical test, LDA: Low dose aspirin, ADPR: adenosine diphosphate receptor I agonist, PDEIII-I: phosphodiesterase III inhibitor, α-GI: α-glucosidase inhibitor, DPP4: dipeptidyl peptidase-4 inhibitor, ACEI: angiotensin coenzyme inhibitor, ARB: angiotensin-2 receptor blocker, Ca-blocker: calcium blocker, NSAIDs: non-steroidal anti-inflammatory drugs.

Among the drugs that were used for the treatment of lifestyle-related diseases, α-GIs (miglitol/voglibose/acarbose: 14/20/2) and statins (rosuvastatin/atorvastatin/pravastatin/pitavastatin/simvastatin/fluvastatin: 20/18/2/2/1/1) were found to be significantly associated with the incidence of colorectal neoplasia. The ratio of the patients who were taking α-GIs in the tumor group was lower than in the non-tumor group (12.9% vs. 27.6%; *p* = 0.023), but the ratio of the patients who were taking statins in the tumor group was significantly higher than in the non-tumor group (35.7% vs. 19.4%; *p* = 0.018). All patients treated with α-GIs (*n* = 36) or statins (*n* = 44) took normal doses of these agents approved as a legitimate treatment in Japan.

### Predictors associated with colorectal neoplasia in patients with type 2 DM

In order to identify the predictors of colorectal neoplasia in patients with type 2 DM, univariate and multivariate analyses were performed with a focus on the candidate variables. In addition to three variables (male gender, α-GI use, and statin use) based on the results shown in Table 2, we selected 11 variables (BMI, hypertension, dyslipidemia, alcohol use, smoking, Biguanide use, ACEI use, ARB use, β-blocker use, LDA use, and NSAIDs use), all of which are reported to increase or decrease the risk of CRC [6, 25, 31–38], for these analyses.

The univariate analysis revealed that male gender (odds ratio [OR] = 2.64, 95% confidence interval [CI] = 1.33–5.24, *p* = 0.005) and taking statins (OR = 2.31, 95% CI = 1.15–4.65, *p* = 0.018) were independent risk factors for colorectal neoplasia. In contrast, taking α-GIs (OR = 0.39, 95% CI = 0.17–0.89, *p* = 0.023) was independently associated with a decreased risk of colorectal neoplasia. The multivariate analysis also revealed that male gender (OR = 4.04, 95% CI = 1.67–10.37, *p* = 0.002) and taking statins (OR = 4.59, 95% CI = 1.69–13.43, *p* = 0.003) were significantly associated with an increased risk, while taking α-GIs (OR = 0.35, 95% CI = 0.13–0.87, *p* = 0.023) and LDA (OR = 0.32, 95% CI = 0.10–0.95, *p* = 0.040) were significantly associated with a decreased risk of colorectal neoplasia in type 2 DM patients (Table 3).

**Table 3:** Predictors of colorectal neoplasia in patients with type 2 DM

	Univariate analysis		Multivariate analysis	
	OR (95% CI)	*p*- value	OR (95% CI)	*p*- value
Age				
< 70 years	1		1	
≥ 70 years	1.18 (0.62–2.26)	0.604	1.70 (0.78–3.82)	0.186
BMI				
< 25 kg/m^2^	1		1	
≥ 25 kg/m^2^	0.76 (0.39–1.48)	0.420	0.79 (0.35–1.75)	0.559
Gender				
female	1		1	
male	2.64 (1.33–5.24)	0.005	4.04 (1.67–10.37)	0.002
Complications				
hypertension	1.06 (0.57–1.93)	0.853	1.37 (0.55–3.44)	0.494
dyslipidemia	1.11 (0.59–2.11)	0.744	0.69 (0.26–1.76)	0.443
Habits				
alcohol use	1.34 (0.70–2.56)	0.376	0.79 (0.33–1.86)	0.582
smoking	1.90 (0.92–3.93)	0.078	1.81 (0.74–4.50)	0.193
Medications				
α-GI	0.39 (0.17–0.89)	0.023	0.35 (0.13–0.87)	0.023
Biguanide	0.89 (0.41–1.94)	0.768	0.70 (0.26–1.84)	0.479
Statin	2.31 (1.15–4.65)	0.018	4.59 (1.69–13.43)	0.003
ACEI	0.69 (0.17–2.84)	0.736	0.63 (0.11–2.88)	0.560
ARB	1.22 (0.64–2.32)	0.546	1.29 (0.52–3.26)	0.582
β-blocker	0.79 (0.22–2.80)	0.764	0.49 (0.10–2.10)	0.340
LDA	0.42 (0.16–1.11)	0.079	0.32 (0.10–0.95)	0.040
NSAIDs	0.98 (0.45–2.09)	0.948	0.89 (0.34–2.25)	0.806

OR: odds ratio, 95% CI: 95% confidence interval, BMI: body mass index, α-GI: α-glucosidase I inhibitor, ACEI: angiotensin coenzyme inhibitor, ARB: angiotensin receptor blocker, LDA: low-dose aspirin, NSAIDs: non-steroidal anti-inflammatory drugs.

## DISCUSSION

Several epidemiological studies have revealed that complications with lifestyle-related diseases, especially type 2 DM, and medications for such diseases are associated with the risk of colorectal tumorigenesis [7]. Among the conventional anti-diabetic medications, the use of metformin by diabetic patients may be associated with a lower risk of CRC [39, 40]. Thiazolidine has also been suggested to exert chemopreventive effects against cancer [41]. In contrast, sulfonylureas, which stimulate insulin secretion, and insulin itself may stimulate cell proliferation and inhibit apoptosis, which may contribute to tumorigenesis [42]. The results of the present study showed the first evidence that taking α-GI may reduce the risk of colorectal tumorigenesis in Japanese type 2 diabetic patients. In a recent nationwide, population-based cohort study in Taiwan, taking acarbose, an α-GI, was found to significantly reduce the risk of CRC development in diabetic patients in a dose-dependent manner [43]. The results were consistent with those of the present study. In addition, the present results showing that taking LDA reduced the incidence of colorectal neoplasia are also consistent with those of previous studies [44].

Several epidemiological and fundamental medical studies have revealed that type 2 DM is as an independent risk factor for CRC [45]. Among the pathophysiological conditions induced by type 2 DM, an increased insulin level is critically involved in colorectal tumorigenesis [11, 46]. These findings also suggest that targeting insulin resistance as well as hyperinsulinemia might be effective strategies for suppressing CRC development [21, 47]. The use of α-GI, which prevents a rapid postprandial increase in the blood glucose level by delaying carbohydrate absorption in the small intestine [48], is reported to improve hyperinsulinemia [49]. This might be a key mechanism underlying its suppression of colorectal neoplasia. A clinical study showed that α-GI therapy reduces the bowel transit time of stool in diabetic patients [50], which also explains the protective effect of α-GI against colorectal tumorigenesis because the long-term exposure to bile acids due to delayed stool transit plays a role in CRC development [51]. α-GI is reported to increase the levels of butyrate, which is associated with the inhibited growth of the transformed cells in the colorectal mucosa [52]. The antineoplastic effects of α-GI, which include the prevention of angiogenesis and the inhibition of tumor growth, have been reported in basic studies [23]. These findings suggest that α-GI may both directly and indirectly suppress colorectal tumorigenesis.

Statins, which are widely used as lipid-lowering drugs, have been shown to safely and effectively reduce mortality from cardiovascular disease [53]. They have also demonstrated antineoplastic properties in various organs, including the colorectum, in experimental studies [28, 54]. Epidemiological studies have suggested the chemopreventive effects of statins against CRC [55]; however, taking statins was found to be an independent risk factor for colorectal neoplasia in the present study. This result might be due to the limitations of the present study. First, this small-size case-control study did not take the administration period of significant drugs, including statins and α-GIs, into account. Second, the serum levels of cholesterol and its density before and after statin treatment were not evaluated. These evaluations are important, as high serum concentrations of high-density lipoprotein cholesterol are associated with a decreased risk of colon cancer [33], but a rapid reduction in cholesterol might increase the risk of colorectal tumorigenesis [56]. The evaluation of the types of statins (i.e. lipophilic or hydrophilic statins) that are prescribed to patients is also significant because lipid solubility is associated with the chemopreventive effects of these agents [57]. Future large-scale prospective studies should be conducted to clarify these limitations.

Finally, it should be emphasized that the early detection of colorectal neoplasia and their prevention are critical to reducing CRC-related mortality [5, 58]. Thus, subjects who have several risk factors for CRC should undergo careful examinations such as TCS [6]. The results of this study may suggest that male diabetic patients taking statins in particular should undergo TCS. Furthermore, intervention trials to clarify the chemopreventive effects of α-GIs on colorectal tumorigenesis seem to be particularly important, as targeting the metabolic alterations associated with type 2 DM, such as hyperinsulinemia, can be an effective strategy for preventing the development of CRC in patients with lifestyle-related diseases [8].

In conclusion, taking α-GIs and/or LDA may be an effective way of suppressing colorectal tumorigenesis in patients with type 2 DM. In contrast, male gender and taking statins may be associated with an increased risk of developing CRC in diabetic patients, and these individuals may require careful surveillance.

## MATERIALS AND METHODS

### Subjects and study protocol

A total of 1176 patients who underwent total colonoscopy (TCS) at Gihoku Kosei Hospital for the first time from August 2012 to June 2014 were enrolled in this retrospective study. These patients were divided by complication with type 2 DM. The exclusion criterion was the presence of ulcerative colitis (UC), as patients diagnosed with this disease are known to be a high-risk group for CRC [59].

The subjects were carefully interviewed prior to TCS to determine their characteristics (age, gender, body mass index [BMI], HbA1c values and the result of fecal immunochemical test), habits (alcohol consumption and smoking), complications with lifestyle-related diseases (hypertension and dyslipidemia) and prescriptions for these diseases (low-dose aspirin [LDA], adenosine diphosphate receptor I agonist [ADPR], phosphodiesterase III inhibitor [PDEIII-I], anti-coagulant, sulfonylureas, tiazoridine, α-glucosidase inhibitor [α-GI], glinide, dipeptidyl peptidase-4 inhibitor [DPP4], insulin, angiotensin coenzyme inhibitor [ACEI], angiotensin-2 receptor blocker [ARB], calcium blocker [Ca-blocker], β-blocker, α-blocker, statin, fibrate and non-steroidal anti-inflammatory drugs [NSAIDs]). All subjects provided their written informed consent before enrollment. The study protocol was approved by the ethics committee of Gihoku Kosei Hospital and performed in accordance with the Declaration of Helsinki.

### Statistical analyses

The χ^2^ test or Student's *t*-test were used to compare the proportions of categorical variables between the diabetic and non-diabetic groups. The correlation between the individual factors (age, gender, BMI, FIT positivity, HbA1c values, complications, habits and current medications) and the prevalence of colorectal neoplasia (CRC and adenoma) was also evaluated by the Pearson χ^2^ test, Student's *t*-test, or Fisher's exact test. A univariate analysis was performed in type 2 DM patients to identify possible predictors of colorectal neoplasia, and a multivariate analysis was then carried out with the variables that were significant in the univariate analysis (*p* < 0.05) and that had been reported to increase or decrease the risk of CRC.

All of the statistical analyses were performed using the JMP^®^ 11 software program (SAS Institute Inc., Cary, NC, USA). *P* values of < 0.05 were considered to indicate statistical significance.

## References

[1] Edwards BK, Noone AM, Mariotto AB, Simard EP, Boscoe FP, Henley SJ, Jemal A, Cho H, Anderson RN, Kohler BA, Eheman CR, Ward EM (2014). Annual Report to the Nation on the status of cancer, 1975-2010, featuring prevalence of comorbidity and impact on survival among persons with lung, colorectal, breast, or prostate cancer. Cancer.

[2] Durko L, Malecka-Panas E (2014). Lifestyle Modifications and Colorectal Cancer. Curr Colorectal Cancer Rep.

[3] Zauber AG, Winawer SJ, O’Brien MJ, Lansdorp-Vogelaar I, van Ballegooijen M, Hankey BF, Shi W, Bond JH, Schapiro M, Panish JF, Stewart ET, Waye JD (2012). Colonoscopic polypectomy and long-term prevention of colorectal-cancer deaths. N Engl J Med.

[4] Giovannucci E (1995). Insulin and colon cancer. Cancer Causes Control.

[5] Tárraga López PJ, Albero JS, Rodríguez-Montes JA (2014). Primary and secondary prevention of colorectal cancer. Clin Med Insights Gastroenterol.

[6] Ohno T, Adachi S, Okuno M, Horibe Y, Goto N, Iwama M, Yamauchi O, Kojima T, Saito K, Ibuka T, Yasuda I, Araki H, Moriwaki H, Shimizu M (2016). Development of a Novel Scoring System for Predicting the Risk of Colorectal Neoplasia: A Retrospective Study. PLoS One.

[7] Guraya SY (2015). Association of type 2 diabetes mellitus and the risk of colorectal cancer: A meta-analysis and systematic review. World J Gastroenterol.

[8] Shirakami Y, Shimizu M, Kubota M, Araki H, Tanaka T, Moriwaki H, Seishima M (2014). Chemoprevention of colorectal cancer by targeting obesity-related metabolic abnormalities. World J Gastroenterol.

[9] Giovannucci E, Harlan DM, Archer MC, Bergenstal RM, Gapstur SM, Habel LA, Pollak M, Regensteiner JG, Yee D (2010). Diabetes and cancer: a consensus report. CA Cancer J Clin.

[10] Yuhara H, Steinmaus C, Cohen SE, Corley DA, Tei Y, Buffler PA (2011). Is diabetes mellitus an independent risk factor for colon cancer and rectal cancer?. Am J Gastroenterol.

[11] Yang YX, Hennessy S, Lewis JD (2004). Insulin therapy and colorectal cancer risk among type 2 diabetes mellitus patients. Gastroenterology.

[12] Corpet DE, Jacquinet C, Peiffer G, Taché S (1997). Insulin injections promote the growth of aberrant crypt foci in the colon of rats. Nutr Cancer.

[13] Tran TT, Medline A, Bruce WR (1996). Insulin promotion of colon tumors in rats. Cancer Epidemiol Biomarkers Prev.

[14] Campbell PT, Deka A, Jacobs EJ, Newton CC, Hildebrand JS, McCullough ML, Limburg PJ, Gapstur SM (2010). Prospective study reveals associations between colorectal cancer and type 2 diabetes mellitus or insulin use in men. Gastroenterology.

[15] Yin S, Bai H, Jing D (2014). Insulin therapy and colorectal cancer risk among type 2 diabetes mellitus patients: a systemic review and meta-analysis. Diagn Pathol.

[16] Derry MM, Raina K, Agarwal C, Agarwal R (2013). Identifying molecular targets of lifestyle modifications in colon cancer prevention. Front Oncol.

[17] Shimizu M, Adachi S, Masuda M, Kozawa O, Moriwaki H (2011). Cancer chemoprevention with green tea catechins by targeting receptor tyrosine kinases. Mol Nutr Food Res.

[18] Shimizu M, Deguchi A, Lim JT, Moriwaki H, Kopelovich L, Weinstein IB (2005). (-)-Epigallocatechin gallate and polyphenon E inhibit growth and activation of the epidermal growth factor receptor and human epidermal growth factor receptor-2 signaling pathways in human colon cancer cells. Clin Cancer Res.

[19] Adachi S, Nagao T, Ingolfsson HI, Maxfield FR, Andersen OS, Kopelovich L, Weinstein IB (2007). The inhibitory effect of (-)-epigallocatechin gallate on activation of the epidermal growth factor receptor is associated with altered lipid order in HT29 colon cancer cells. Cancer Res.

[20] Guraya SY (2014). Chemopreventive role of vitamin D in colorectal carcinoma. Journal of Microscopy and Ultrastructure.

[21] Shimizu M, Shirakami Y, Sakai H, Adachi S, Hata K, Hirose Y, Tsurumi H, Tanaka T, Moriwaki H (2008). (-)-Epigallocatechin gallate suppresses azoxymethane-induced colonic premalignant lesions in male C57BL/KsJ-db/db mice. Cancer Prev Res (Phila).

[22] Shimizu M, Fukutomi Y, Ninomiya M, Nagura K, Kato T, Araki H, Suganuma M, Fujiki H, Moriwaki H (2008). Green tea extracts for the prevention of metachronous colorectal adenomas: a pilot study. Cancer Epidemiol Biomarkers Prev.

[23] Pili R, Chang J, Partis RA, Mueller RA, Chrest FJ, Passaniti A (1995). The alpha-glucosidase I inhibitor castanospermine alters endothelial cell glycosylation, prevents angiogenesis, and inhibits tumor growth. Cancer Res.

[24] Viollet B, Guigas B, Sanz Garcia N, Leclerc J, Foretz M, Andreelli F (2012). Cellular and molecular mechanisms of metformin: an overview. Clin Sci (Lond).

[25] Higurashi T, Hosono K, Takahashi H, Komiya Y, Umezawa S, Sakai E, Uchiyama T, Taniguchi L, Hata Y, Uchiyama S, Hattori A, Nagase H, Kessoku T (2016). Metformin for chemoprevention of metachronous colorectal adenoma or polyps in post-polypectomy patients without diabetes: a multicentre double-blind, placebo-controlled, randomised phase 3 trial. Lancet Oncol.

[26] Makar GA, Holmes JH, Yang YX (2014). Angiotensin-converting enzyme inhibitor therapy and colorectal cancer risk. J Natl Cancer Inst.

[27] Jung YS, Park CH, Eun CS, Park DI, Han DS (2016). Statin use and the risk of colorectal adenoma: A meta-analysis. J Gastroenterol Hepatol.

[28] Yasuda Y, Shimizu M, Shirakami Y, Sakai H, Kubota M, Hata K, Hirose Y, Tsurumi H, Tanaka T, Moriwaki H (2010). Pitavastatin inhibits azoxymethane-induced colonic preneoplastic lesions in C57BL/KsJ-db/db obese mice. Cancer Sci.

[29] Kochi T, Shimizu M, Ohno T, Baba A, Sumi T, Kubota M, Shirakami Y, Tsurumi H, Tanaka T, Moriwaki H (2014). Preventive effects of the angiotensin-converting enzyme inhibitor, captopril, on the development of azoxymethane-induced colonic preneoplastic lesions in diabetic and hypertensive rats. Oncol Lett.

[30] Seino Y, Nanjo K, Tajima N, Kadowaki T, Kashiwagi A, Araki E, Ito C, Inagaki N, Iwamoto Y, Kasuga M, Hanafusa T, Haneda M, Ueki K, Committee of the Japan Diabetes Society on the Diagnostic Criteria of Diabetes Mellitus (2010). Report of the committee on the classification and diagnostic criteria of diabetes mellitus. J Diabetes Investig.

[31] Renehan AG, Tyson M, Egger M, Heller RF, Zwahlen M (2008). Body-mass index and incidence of cancer: a systematic review and meta-analysis of prospective observational studies. Lancet.

[32] Radišauskas R, Kuzmickienė I, Milinavičienė E, Everatt R (2016). Hypertension, serum lipids and cancer risk: A review of epidemiological evidence. Medicina (Kaunas).

[33] van Duijnhoven FJ, Bueno-De-Mesquita HB, Calligaro M, Jenab M, Pischon T, Jansen EH, Frohlich J, Ayyobi A, Overvad K, Toft-Petersen AP, Tjønneland A, Hansen L, Boutron-Ruault MC (2011). Blood lipid and lipoprotein concentrations and colorectal cancer risk in the European Prospective Investigation into Cancer and Nutrition. Gut.

[34] Botteri E, Iodice S, Raimondi S, Maisonneuve P, Lowenfels AB (2008). Cigarette smoking and adenomatous polyps: a meta-analysis. Gastroenterology.

[35] Rothwell PM, Wilson M, Elwin CE, Norrving B, Algra A, Warlow CP, Meade TW (2010). Long-term effect of aspirin on colorectal cancer incidence and mortality: 20-year follow-up of five randomised trials. Lancet.

[36] Kedika R, Patel M, Pena Sahdala HN, Mahgoub A, Cipher D, Siddiqui AA (2011). Long-term use of angiotensin converting enzyme inhibitors is associated with decreased incidence of advanced adenomatous colon polyps. J Clin Gastroenterol.

[37] Algazi M, Plu-Bureau G, Flahault A, Dondon MG, Lê MG (2004). [Could treatments with beta-blockers be associated with a reduction in cancer risk?]. Rev Epidemiol Sante Publique.

[38] Smalley W, Ray WA, Daugherty J, Griffin MR (1999). Use of nonsteroidal anti-inflammatory drugs and incidence of colorectal cancer: a population-based study. Arch Intern Med.

[39] Libby G, Donnelly LA, Donnan PT, Alessi DR, Morris AD, Evans JM (2009). New users of metformin are at low risk of incident cancer: a cohort study among people with type 2 diabetes. Diabetes Care.

[40] Currie CJ, Poole CD, Gale EA (2009). The influence of glucose-lowering therapies on cancer risk in type 2 diabetes. Diabetologia.

[41] Okumura T (2010). Mechanisms by which thiazolidinediones induce anti-cancer effects in cancers in digestive organs. J Gastroenterol.

[42] Bowker SL, Majumdar SR, Veugelers P, Johnson JA (2006). Increased cancer-related mortality for patients with type 2 diabetes who use sulfonylureas or insulin. Diabetes Care.

[43] Tseng YH, Tsan YT, Chan WC, Sheu WH, Chen PC (2015). Use of an α-Glucosidase Inhibitor and the Risk of Colorectal Cancer in Patients With Diabetes: A Nationwide, Population-Based Cohort Study. Diabetes Care.

[44] Cole BF, Logan RF, Halabi S, Benamouzig R, Sandler RS, Grainge MJ, Chaussade S, Baron JA (2009). Aspirin for the chemoprevention of colorectal adenomas: meta-analysis of the randomized trials. J Natl Cancer Inst.

[45] Yang YX, Hennessy S, Lewis JD (2005). Type 2 diabetes mellitus and the risk of colorectal cancer. Clin Gastroenterol Hepatol.

[46] Keku TO, Lund PK, Galanko J, Simmons JG, Woosley JT, Sandler RS (2005). Insulin resistance, apoptosis, and colorectal adenoma risk. Cancer Epidemiol Biomarkers Prev.

[47] Shimizu M, Shirakami Y, Iwasa J, Shiraki M, Yasuda Y, Hata K, Hirose Y, Tsurumi H, Tanaka T, Moriwaki H (2009). Supplementation with branched-chain amino acids inhibits azoxymethane-induced colonic preneoplastic lesions in male C57BL/KsJ-db/db mice. Clin Cancer Res.

[48] Göke B, Fuder H, Wieckhorst G, Theiss U, Stridde E, Littke T, Kleist P, Arnold R, Lücker PW (1995). Voglibose (AO-128) is an efficient alpha-glucosidase inhibitor and mobilizes the endogenous GLP-1 reserve. Digestion.

[49] Inoue I, Takahashi K, Noji S, Awata T, Negishi K, Katayama S (1997). Acarbose controls postprandial hyperproinsulinemia in non-insulin dependent diabetes mellitus. Diabetes Res Clin Pract.

[50] Ron Y, Wainstein J, Leibovitz A, Monastirsky N, Habot B, Avni Y, Segal R (2002). The effect of acarbose on the colonic transit time of elderly long-term care patients with type 2 diabetes mellitus. J Gerontol A Biol Sci Med Sci.

[51] Citronberg J, Kantor ED, Potter JD, White E (2014). A prospective study of the effect of bowel movement frequency, constipation, and laxative use on colorectal cancer risk. Am J Gastroenterol.

[52] Weaver GA, Tangel CT, Krause JA, Parfitt MM, Stragand JJ, Jenkins PL, Erb TA, Davidson RH, Alpern HD, Guiney WB, Higgins PJ (2000). Biomarkers of human colonic cell growth are influenced differently by a history of colonic neoplasia and the consumption of acarbose. J Nutr.

[53] Fulcher J, O’Connell R, Voysey M, Emberson J, Blackwell L, Mihaylova B, Simes J, Collins R, Kirby A, Colhoun H, Braunwald E, La Rosa J, Pedersen TR, Cholesterol Treatment Trialists’ (CTT) Collaboration (2015). Efficacy and safety of LDL-lowering therapy among men and women: meta-analysis of individual data from 174,000 participants in 27 randomised trials. Lancet.

[54] Lochhead P, Chan AT (2013). Statins and colorectal cancer. Clin Gastroenterol Hepatol.

[55] Cai H, Zhang G, Wang Z, Luo Z, Zhou X (2015). Relationship between the use of statins and patient survival in colorectal cancer: a systematic review and meta-analysis. PLoS One.

[56] Mamtani R, Lewis JD, Scott FI, Ahmad T, Goldberg DS, Datta J, Yang YX, Boursi B (2016). Disentangling the Association between Statins, Cholesterol, and Colorectal Cancer: A Nested Case-Control Study. PLoS Med.

[57] Liu Y, Tang W, Wang J, Xie L, Li T, He Y, Deng Y, Peng Q, Li S, Qin X (2014). Association between statin use and colorectal cancer risk: a meta-analysis of 42 studies. Cancer Causes Control.

[58] Winawer SJ, Zauber AG, Ho MN, O’Brien MJ, Gottlieb LS, Sternberg SS, Waye JD, Schapiro M, Bond JH, Panish JF, Ackroyd F, Shike M, Kurtz RC, The National Polyp Study Workgroup (1993). Prevention of colorectal cancer by colonoscopic polypectomy. N Engl J Med.

[59] Farraye FA, Odze RD, Eaden J, Itzkowitz SH (2010). AGA technical review on the diagnosis and management of colorectal neoplasia in inflammatory bowel disease. Gastroenterology.

